# First-Person Neuroscience: A new methodological approach for linking mental and neuronal states

**DOI:** 10.1186/1747-5341-1-3

**Published:** 2006-03-17

**Authors:** Georg Northoff, Alexander Heinzel

**Affiliations:** 1 Department of Psychiatry, University of Magdeburg, Magdeburg, Germany; 2 Department of Nuclear Medicine, University of Duesseldorf, Duesseldorf, Germany

## Abstract

Though the brain and its neuronal states have been investigated extensively, the neural correlates of mental states remain to be determined. Since mental states are experienced in first-person perspective and neuronal states are observed in third-person perspective, a special method must be developed for linking both states and their respective perspectives. We suggest that such method is provided by First-Person Neuroscience. What is First-Person Neuroscience? We define First-Person Neuroscience as investigation of neuronal states under guidance of and on orientation to mental states. An empirical example of such methodological approach is demonstrated by an fMRI study on emotions. It is shown that third- and first-person analysis of data yield different results. First-person analysis reveals neural activity in cortical midline structures during subjective emotional experience. Based on these and other results neural processing in cortical midline structures is hypothesized to be crucially involved in generating mental states. Such direct linkage between first- and third-person approaches to analysis of neural data allows insight into the "point of view from within the brain", that is what we call the First-Brain Perspective. In conclusion, First-Person Neuroscience and First-Brain Perspective provide valuable methodological tools for revealing the neuronal correlate of mental states.

## Introduction

We experience events in the environment. We are conscious of some of the events we experience. We thus experience mental states in first-person perspective. In contrast, we never observe mental states in other persons and thus in third-person perspective. Instead of other persons' mental states, we can observe their brains with its neuronal states. Due to this dissociation between first- and third-person perspective, mental and neuronal states have often been considered separately and isolated from each other ultimately resulting in dualism between brain and mind [1]. It should be noted that we do not intend to imply that this description reflects the historical development in the philosophy of mind. From a historical point of view the development might be considered as vice versa (see e.g. [2] for a more detailed discussion).

However, the modern imaging techniques allow new insights and on-line access to the brain while experiencing mental states in first-person perspective. In order to reveal the true neuronal correlates of mental states, first- and third-person perspective must be linked to each other. Such linkage requires a special methodological approach where both perspectives are directly related to each other. This has been discussed recently under the heading of "first-person methodologies" [3,4]. Here we want to present an outline of such an approach with respect to neuronal states, First-Person Neuroscience.

In a first section definition and concept of First-Person Neuroscience are presented. This is followed by demonstration of an empirical example, an own fMRI study on emotions, where both approaches first- and third-person are directly compared to each other. The results reveal the crucial role of neural processing in cortical midline structures in generating emotional mental states. We here focus on emotional mental states. Therefore it remains unclear to what extend this also applies to mental states in general as well to the distinct features of mental states. In the final section, epistemic implications of such First-Person Neuroscience shall be discussed. We suggest that First-Person Neuroscience presupposes a "point of view from within the brain" which we call First-Brain Perspective.

## First-Person Neuroscience: Definition and concept

### What is First-Person Neuroscience?

**Figure 1:**
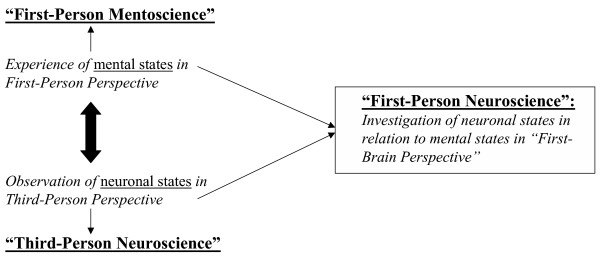
Linkage between mental and neuronal states in "First-Person Neuroscience".

First-Person Neuroscience uses methods for the systematic examination and evaluation of mental states by themselves and their contents as experienced in first-person perspective and links them with data about neuronal states as obtained in third-person perspective (Figure 1). Such methods include, for example, phenomenology and introspective psychology that may be regarded as steps towards the development of a "science of experience" (see also [5] for the distinction between introspection and phenomenological analysis). Linkage between first- and third-person data in First-Person Neuroscience depends on and thus presupposes a reliable and detailed account of the first-person data by themselves and thus a 'science of experience'. The better the first-person data are accounted for the better and more promising their linkage with third-person data: "Thus, for example, a large-scale integration mechanism in the brain such as neural synchrony in the gamma band should be validated also on the basis of its ability to provide insight into first-person accounts of mental contents such as duration. The empirical questions must be guided by first-person evidence" [5]; (see also [1,3,6-8]). This, however, contrasts with most current empirical approaches that investigate the brain only in third-person perspective. Empirical investigations of brain states focus exclusively on neuronal states and presuppose therefore only the third-person perspective. This approach may subsequently be called Third-Person Neuroscience.

Third-Person Neuroscience can be defined by empirical investigations of brain states in the third-person perspective. It is therefore necessarily restricted to neuronal states and cannot account for subjective mental states, which remain inaccessible in third-person perspective. First-Person Neuroscience, in contrast, can be defined by the empirical investigation of brain states in orientation to systematic epistemic linkage between First- and third-person perspective; this accounts for empirical linkage between mental and neuronal states. As a result First-Person Neuroscience focuses on the development of methods for the systematic linkage between First- and third-person data: "Third, it would be futile to stay with first-person descriptions in isolation. We need to harmonize and constrain them by building the appropriate links with third-person studies. .... To make this possible we seek methodologies that can provide an open link to objective, empirically based description." [3].

Observation in third-person perspective, as presupposed in physics and Third-Person Neuroscience, remains insufficient by itself in the empirical investigation of the mental states since the latter can be accessed only by experience and thus in First-Person Perspective. This has already been pointed out by Hume who, relying on one of his current interpreters emphasized the role of introspection: "Hume sought to adapt the experimental method of Newton to the investigation of the powers and principles of the human mind launched by Locke. Here we have said "adapt" rather than "adopt", because Hume did not think that physical experiments could be performed on the mind. Rather, he thought that the mind's workings are accessible to introspection, and that by careful introspective study of one's own conscious states, one would be able to discover general principles that apply to those states; much as by carefully studying the operations of physical objects Newton had discovered general principles applying to them, such as the laws of motion and gravitation. The result of this essentially introspective study of the mind was to be a truly empirical science of human nature" [9].

Often, methods for evaluation of mental states and those for their linkage to third-person data have been subsumed under the term "First-Person Methodologies" [3]. Moreover, First-Person Neuroscience is often equated with Second-Person Neuroscience. A Second-Person Neuroscience focuses on those mental states that can be detected in second-person perspective by means of introspection or "phenomenal judgment" [1]. For example, investigation of neural correlates of consciousness may be considered a paradigmatic example of Second-Person Neuroscience or "neurophenomenology" [5]. It should be noted that the term "phenomenal" includes both conscious and unconscious states in order to account for the full range of mental states as experienced and judged in First- and Second-Person Perspective. Despite these differences in the range of mental states, Second-Person Epistemology is often not differentiated from First-Person Neuroscience (see, for example, [5,6]). In the following use of the term "First-Person Neuroscience", Second-Person Epistemology is included for pragmatic purposes. It should also be noted that we presuppose a rather broad meaning of the term "neuroscience". It includes all disciplines involved in the direct or indirect empirical investigation of the brain ranging from psychology over neurocomputation to neurogenetics. However, not all mental states, as experienced in first-person perspective, are conscious and can consequently be detected and recognized in second-person perspective. Second-Person Neuroscience is therefore not necessarily identical to First-Person Neuroscience since the latter covers a broader spectrum of mental i.e. unconscious and conscious states than the former which remains restricted to conscious states. An example of 'First-Person Neuroscience' as distinguished from 'Second-Person Neuroscience' consists in the investigation of the neural states underlying psychodynamic and thus unconscious processes. For example, certain psychodynamic parameters, which were altered in patients with catatonic schizophrenia, correlated significantly with deactivation in orbitofrontal cortex during emotional stimulation [7,10,11].

### How can First-Person Neuroscience be implemented in empirical research?

First-Person Neuroscience provides the linkage between mental and neuronal states. Methodologically, the linkage between neuronal and mental states is provided by "disciplined circularity" or a so-called "neuro-phenomenological circulation" [5] between neuronal and mental states where both can be considered as "mutual or reciprocal constraints" for each other in neuroscientific investigation. This, in turn, provides indirect access to the neural correlates of mental states. There may be different methodological strategies for linking neuronal and mental states in an empirical investigation.

First, the contents of mental states can be related to neuronal states. For example, different types of emotions i.e. positive, negative, disgust, happiness, etc. may be related to distinct spatio-temporal activation patterns in the prefrontal cortex [12-14]. However, the problem that arises here is that the contents, as determined and categorized in third-person perspective for empirical investigations of mental states, may not necessarily be identical with the ones as experienced in first-person perspective.

Second, the neuronal states underlying the subjective experience of mental states may indirectly be accounted for by the combination of different methods of analysis. One such methodological approach that provides linkage between First- and third-person perspective in empirical investigation of brain states, can be characterized as "double analysis". In "double analysis", the same data are analyzed with regard to both objectively and subjectively correct answers. For example, subjects have to decide whether certain presented items are blue or black. They may make correct decisions about the color of these items. In addition, they may make some mistakes by pointing out the wrong color. Data about neuronal states, obtained during the process of decision, may be grouped and analyzed in two different ways. First, all items classified correctly as blue, may be compared with those classified correctly as black. This type of analysis would be a Third-Person analysis since only items classified as objectively correct, according to third-person perspective, are grouped together. Second, all items classified as blue (thus also including the ones wrongly classified as blue) may be compared with all those classified as black (thus also including the ones wrongly classified as black). This type of analysis would be a First-Person analysis since all items classified as subjectively correct, according to first-person perspective, are grouped together. Results from both types of analysis may be compared with each other. Differences between the results from both analyses may reflect the difference between subjective and objective classification and thus between first- and third-person perspective.

Third, different characteristics of the subjective experience itself may serve as a guide and orientation for the analysis of data regarding neuronal states. The characteristics of subjective experiences may be revealed in a so-called "phenomenological analysis" [5]. Relying on "introspection" and "phenomenological analysis", a so-called "phenomenological cluster" [6] may be elucidated. For example, different "phenomenological clusters" were revealed by means of subjective questioning i.e. introspection during the time course of visual illusions. These different temporal "phenomenological clusters" then served as the guide for analysing different brain rhythms in the respective "subjective" time intervals. Different time intervals and thus different "phenomenological clusters" could indeed be characterized by different brain rhythms (theta, alpha, beta, gamma) [6]. Another example of such a "phenomenological analysis" would be the analysis of fMRI data in orientation to the phenomenological concepts of temporality i.e. "phenomenal time" [15]. Lloyd observed that the multivariate distance and changes between brain images is approximately linearly related to their temporal distance. The more closer acquired in time the more similar the images. Thus, the changes between the different images occur gradually over time. Lloyd argues that these results are consistent with Husserl's description of time consciousness in that they reflect the inexorable temporal flux of the conscious state. Analogous to the way that each moment of our phenomenological experience of time builds on foundation of the previous moment, the series of fMRI images appears to form a continuously evolving temporal pattern of global activity.

## First- and Third-Person Neuroscience: Empirical example

### How can neuronal states be investigated in first- and third-person perspective?

We want to present an empirical example for the comparison between a first- and third-person approach to investigation of neuronal states, an fMRI study on emotions [16]. It should however be noted that emotional experience presents a special case of mental states. Thus it remains unclear to what extend these results may be generalised to other mental states.

Another important distinction in this context is Ryle's distinction between episodic and dispositional mental states with the former being timed and introspectable while the latter cannot be timed off/on and infallibly introspected [17]. What we here focus on are episodic mental states, the mental states in relation to the presented emotional pictures. It may however be the case that the regions discussed below, the cortical midline structures, could also underlie our continuous dispositions for certain mental states and thus what Ryle calls dispositional mental states.

Based on a well validated affective picture system (International Affective Picture System; IAPS) emotions can be classified into positive, neutral, and negative categories by the investigator – this can be called third-person or categorical approach. The investigation of the neural correlates of these positive, negative, and neutral emotional categories can subsequently be characterized as Third-Person Neuroscience. Here the neural correlates, as for example obtained with fMRI, are related to the emotional categories as determined by the third-person perspective of the investigator.

However, emotions must be considered as special case since they are inherently subjective. Even the classification of emotions into positive, negative, and neutral categories relies ultimately on subjective continuous ratings in first-person perspective, performed by those subjects which served as the basis for determination of the standard valence scale. Consequently, investigation of the neuronal basis of the different emotional categories (positive, negative, neutral) combine data of both first- and third-person neuronal correlates.

How can we separate the neuronal correlates belonging to continuous emotional experience in first-person perspective from those associated with categorical distinction of emotions in third-person perspective? First-Person Neuroscience provides a special methodological approach for elucidation of the first-person neuronal correlates. One can classify emotions not only according to the third-person categories of the investigator but also with respect to the continuous first-person experience of the investigated subjects. The continuous first-person experience can for example be obtained on a visual analogue scale with a continuum between 1 and 9 of emotional valences – this continuous or parametric analysis can be called first-person approach. In this case, the first-person experience of the emotions can directly be related to the neuronal correlates as measured in fMRI. Such a direct relation is possible since the emotional experience has been transformed via a visual analogue scale into numerical values that can be correlated with the values resulting from the fMRI measurement. It has to be considered that the numerical values are not identical with the emotional experience itself. In a next step one might then compare the fMRI results from both approaches, the categorical or third-person versus the parametric or first-person analysis. If both analyses reveal different regions, the neuronal correlates of third-person categorization of emotions can be separated from the first-person (parametric) experience. As such the neuronal correlates specifically underlying first-person experience and thus mental states can be revealed and separated from those associated with third-person observation.

### What are the differences between first- and third-person analysis of emotions?

In an fMRI study (13 subjects) on visual experience (IAPS) of emotional pictures [14,16], we compared both approaches, the parametric first-person and the categorical third-person approach.

Two types of statistical analyses of the fMRI data were performed. In a first step, we adopted a categorical analysis. The constructed regressors were the presented pictures, irrespective of their emotional valence. The main purpose of this analysis was to detect the regions responsible for neural processing during the presentation of the IAPS pictures in general.

In a second step, we adopted a parametric analysis using the valence ratings (1–9) as modulation parameter. As in the first step it was tested against baseline. The valence values used for this analysis were taken from the SAM ratings of our subjects. This was to assure that the appropriate (i.e., individually rated) valence values were applied to the analysis for each subject.

The analysis tested for a linear relationship between regional signal changes and valences of IAPS pictures and thus valence-dependent modulation of signal intensity in particular regions.

The linear relationship of the changes in the BOLD signal with the valence values was further illustrated by showing the regressions for local maxima of the BOLD signal from the parametric analysis.

**Figure 2:**
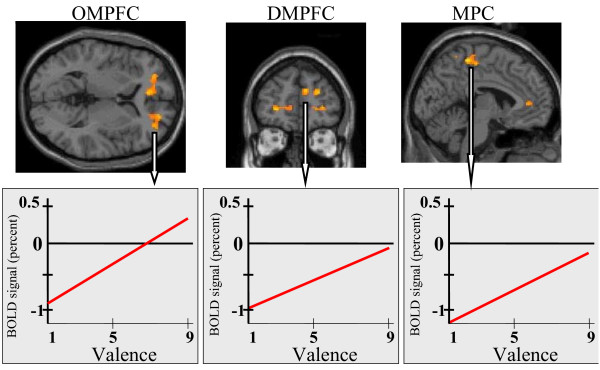
**Parametric or first-person modulation in cortical midline regions during emotional picture presentation**. **a) **Upper part. FMRI images represent results from a random effects group analysis (n = 13) depicted on a standard MNI brain. X, y, z: MNI coordinates in mm. X describes right (+)/left (-), y anterior (+)/posterior (-), and z superior (+)/inferior (-) distances. Z = Z-score. Only regions with Z > 3.29 (p < 0.001, uncorrected, voxel level) are described. The aim of the analysis was to characterise the parametric modulation of the BOLD-signal during the presentation of IAPS-pictures. To that end we adopted a parametric analysis using the valence ratings (1–9 rated by the subjects in a post hoc analysis) as the modulation parameter, which was tested against baseline. Parametric modulations of regional signal intensities during presentation of IAPS pictures were obtained in OMPFC (x = 16, y = 56, z = 19; Z = 3.35), DMPFC (x = 12, y = 48, z = 24; Z = 3.29; visible in the coronal image), MPC (x = -2, y = 34, z = 60; Z = 3.68) and left insula (x = -54, y = -10, z = 14; Z = 4.3). **b) **Lower Part. The parametric relationship between picture valence (x-axis, valence values from 1 [negative] to 9 [positive]) and signal percent change (y-axis, intensity change of the BOLD signal) during viewing of IAPS pictures is demonstrated. The regional local maxima of valence-dependent modulation were correlated with the picture valences. Parametric valence-dependent modulation of signal percent change was found in the following regions during IAPS picture viewing: The OMPFC (x = 16, y = 56, z = 19; Z = 3.35), the DMPFC (x = 12, y = 48, z = 24; Z = 3.29) and the MPC (x = -2, y = -34, z = 60; Z = 3.68). OMPFC = orbitomedial prefrontal cortex, DMPFC = dorsomedial prefrontal cortex, MPC = medial parietal cortex.

And indeed we obtained different results in both analyses. The third-person approach revealed several regions across the whole cortex whereas the first-person approach showed only regions in the cortical midline, the orbitomedial (OMPFC) and dorsomedial (DMPFC) prefrontal cortex and the medial parietal cortex (MPC) (Table 1 and Figure 2; see [16] for further details).

**Table 1:** Signal changes as obtained in categorical or third-person and parametric or first-person analysis.

	Signal changes in categorical analysis (third-person)	Signal changes in parametric analysis (first-person)
Orbitomedial prefrontal cortex (OMPFC)	-2/54/14, 3.65	16/56/19, 3.35
Dorsomedial prefrontal cortex (DMPFC)	4/48/40, 3.68	12/48/24, 3.29
Medial parietal cortex (MPC)	-	-2/-34/60, 3.68
Insula	-	-54/-10/14, 4.32
Bilateral posterior parietal cortex	-48/-67/28, 3.36; 50/-63/25, 3.34	-
Thalamus/Hypothalamus	0/-8/4, 3.39	-
Posterior cingulate	0/-38/40, 3.83	-

X, y, z: MNI coordinates in mm. X describes right (+)/left (-), y anterior (+)/posterior (-), and z superior (+)/inferior (-) distances (e.g. 21/23/43, 3.45 ≅ x = 21/y = 23/z = 43, Z = 3.45). Coordinates of the local maxima of regional signal increases are given. Z = Z-score. Only signal increases with Z > 3.29 (p < 0.001, uncorrected, voxel level) are described.

Signal changes associated with categorical analysis reflect the comparison between the IAPS picture categories and the baseline condition. (Left column)

In contrast, the parametric analysis tested for a linear relationship between regional changes in the BOLD signal and valence of the presented IAPS pictures as rated by the investigated subjects. The IAPS pictures were modeled as a regressor according to their valence ranging from 1 to 9. The parametric signal changes were tested against the baseline condition. Regions are listed that show a positive linear relationship between valence and BOLD signal changes. (Right column)

Note the overlap between both comparisons with regard to involvement of OMPFC and DMPFC. The other regions, in contrast, were either involved in categorical or parametric analysis.

The first step, the categorical analysis, revealed multiple significant foci of signal change in the comparison of IAPS picture viewing with our baseline condition. Significant signal changes were observed in cortical midline regions such as the OMPFC (x = -2, y= 54, z = 14; Z = 3.65) and the DMPFC (x = 4, y = 48, z = 40; Z = 3.68) (Table 1). Moreover, significant signal increases were observed in the bilateral posterior parietal cortex (x = -48, y = -67, z = 28; Z = 3.36) (x = 50, y = -63, z = 25; Z = 3.34), the posterior cingulate (x = 0, y = -38, z = 40; Z = 3.83) and the thalamus/hypothalamus (x = 0, y = -8, z = 4; Z = 3.39) (Table 1).

The second step, the parametric analysis, tested for a positive correlation between the changes of the valence and the linear change of the BOLD signal. It revealed valence-dependent modulation of signal intensity in the OMPFC (x = 16, y = 56 and z = 19; Z = 3.35; close to the pregenual anterior cingulate) and the DMPFC (x = 12, y = 48 and z = 24; Z = 3.29). Other regions showing valence-dependent modulation included signal increases in the MPC (x = -2, y = -34, z = 60; Z = 3.68) and the left insula (x = -54, y = -10, z = 14; Z = 4.32). It should be noted that both comparisons overlap with regard to the involvement of the OMPFC and DMPFC. The other regions, in contrast, were either involved in categorically- or parametrically-indnuced signal changes. The posterior cingulate showed signal changes only in the categorical analysis, whereas involvement of the medial parietal cortex was revealed only in the parametric analysis (Figure 2).

Accordingly, it seems that as if there are regions in the brain, the so-called cortical midline structures [18], which are specifically related to the first-person experience of emotions. More generally, these regions have been assumed to be involved in any type of first-person experience and thus in mental states since they seem to preferentially process self-referential stimuli as distinguished from non-self-referential ones [18].

This example demonstrates that the linkage between first- and third-person perspective can yield additional information, i.e., that cortical midline regions seem to be associated specifically with first-person experience. This information is not provided by mere third-person approaches to emotions where the first-person neuronal correlates cannot be isolated from the ones underlying third-person observation. Methodological strategies for the linkage between first- and third-person data about mental and neuronal states need to be more refined and developed in the future. It can be expected that they will provide us with insight into the neuronal correlates of mental states. Based on my own and others results (see [18] for an overview), we hypothesize that cortical midline structures are crucial in generating subjective experience of mental states.

## First-Person Neuroscience: First-Person Perspective and First-Brain Perspective

### What is the First-Brain Perspective?

**Figure 3:**
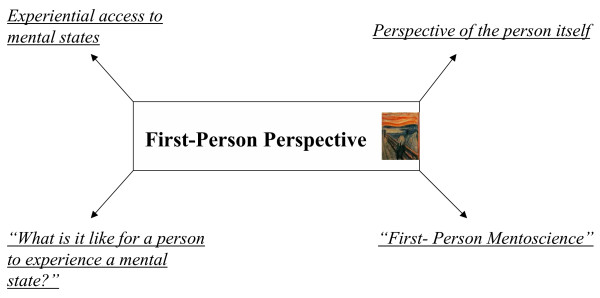
First-Person Perspective.

**Figure 4:**
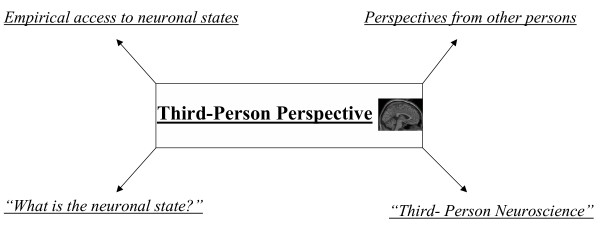
Third-Person Perspective.

**Figure 5:**
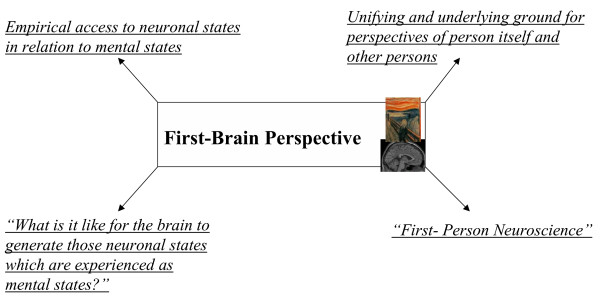
First-Brain Perspective.

In philosophy, experience of mental states in first-person perspective has been characterized by "What is it like for a person (or bat) to experience (or generate) that particular (mental) state?" [19] (see also Figure 3) whereas observation of neuronal states in third-person perspective can be described by "What is the neuronal state?" (see also Figure 4). The first-person perspective reveals insight into the experience of mental states whereas the third-person perspective provides access to observation of neuronal states. Either perspective however does not consider the respective other state, mental states are ignored in third-person perspective whereas neuronal states are neglected in first-person perspective. If one combines both, one might access one's own brain in first-person perspective. This reveals those neuronal states that are experienced as mental states in first-person perspective. One can consequently speak of a First-Brain Perspective [1] and formulate the corresponding "What is it like" sentence: "What is it like for the brain to generate those neuronal states which are experienced as mental states?" (see also Figure 5).

Scientific investigation of mental states in isolation from neuronal states in what may be called First Person Mentoscience (see [1] for details). First Person Mentoscience presupposes the first person perspective as characterized by mental states. The mental states, associated with first person perspective, however are investigated from third person perspective. One thus gets an objective account of subjective or first person states, i.e., of mental states in a scientific way resulting in what we call First Person Mentoscience. Nagel also speaks of an "objective phenomenology" [[20], see also [21]]. In contrast scientific investigation of neuronal states in Third-Person Neuroscience presupposes the third-person perspective. Which perspective is presupposed in First-Person Neuroscience? As demonstrated, First-Person Neuroscience provides linkage between neuronal and mental states and thus between third- and first-person perspective. This allows access though indirectly to the brain itself, i.e., what is it like for the brain to generate those neuronal states which are experienced as mental states in first-person perspective. This presupposes a point of view "from the inside of the brain", i.e., "viewing the brain from within" [22]. We call this point of view First-Brain Perspective. Since this point of view is presupposed, First-Person Neuroscience can be characterized by the First-Brain Perspective. The First-Brain Perspective considers neuronal states in orientation to mental states, i.e., it allows insight into the brains' neuronal states in relation to mental states.

### Why is First-Person Neuroscience necessary?

We have no direct access to our own brain as a brain because we do not experience our own neuronal states as neuronal states in first-person perspective. Instead, we experience mental states in first-person perspective. Mental states in first-person perspective appear thus independent of the brains' neuronal states as observed in third-person perspective. Scientific investigation of mental states as experienced in first-person perspective might thus be independent of observation of neuronal states in third-person perspective – one might thus speak of First-Person Mentoscience [1].

Due to the dissociation between mental and neuronal states with respect to first- and third-person perspective, both types of states have been investigated independently of each other. Subsequently, "neuronal/physical constituents and mental constituents" were distinguished from each other: "Physical phenomena can be analyzed into their physical constituents, with the aid of scientific experimentation, and mental phenomena can perhaps be analyzed into their mental constituents at least in some cases, but these two path of analyses do not meet" [23]. In other terms, First-Person Mentoscience and Third-Person Neurocience remained independent of each other resulting in methodological dualism with isolation between neuronal and mental states.

First-Person Neuroscience attempts at overcoming and bridging such methodological dualism between First-Person Mentoscience and Third-Person Neurocience. This is made possible by the complementary and mutually exclusive abilities in First- and third-person perspective. In first-person perspective, we can experience our own mental states but have no access to our own neuronal states – this inability can be called "autoepistemic limitation" [1]. Whereas in third-person perspective, we can observe others' neuronal states but have no access to their mental states – this inability can be called "heteroepistemic limitation" [1]. First-Person Neuroscience links both first- and third-person perspective and can therefore relate mental states to neuronal states. To answer the question heading this section: First-Person Neuroscience is necessary because there is "autoepistemic limitation" in first-person perspective (and "heteroepistemic limitation" in third-person perspective). As such First-Person Neuroscience provides the methodological tools for compensating our inability to directly experience our own neuronal states as neuronal states in first-person perspective. It provides an escape from "autoepistemic limitation" by offering a method for indirect access to the neuronal states of our own brain.

### Conclusions and conceptual considerations

First-Person Neuroscience provides a methodological approach that can contribute to a better understanding of mental states. By linking first- and third-person perspective, it may help to overcome the dualism between the two perspectives. We suggest that this epistemic dualism might be resolved by developing a new perspective, First-Brain Perspective, which unifies and entails the specific properties of first- and third-person perspective. Though in this paper, we mainly focus on the empirical implications of such first brain perspective, i.e., the methodological approach of First Person Neuroscience, we at least want to indicate some philosophical issues.

It may for example be argued that first-person perspective and third-person perspective constitute conceptual contradictions. Development of a unifying perspective, such as first brain perspective is then impossible for conceptual reasons (e.g. [24]) resulting in what may be called epistemic dualism. Other argued that mental phenomena per se are part of a wrong theory of mind, which will be replaced by future results of empirical neuroscience (e.g. [25,26]. In this case there is no need to postulate any first perspective be it related to a person, i.e., first-person perspective, or to the brain, i.e., First-Brain Perspective. Therefore the eliminative materialism may be considered as an epistemic monism.

What is common to epistemic dualism and monism is that they make the assumption of First-Brain Perspective impossible, either for being conceptually contradictory, as in epistemic dualism, or for being empirically superfluous as in eliminative materialism. It is therefore clear that our approach that postulates a First-Brain Perspective must undermine the alternative between epistemic dualism and monism by taking an intermediary stance. However, what remains unclear is how such an intermediary stance might look like. For example, it has to be discussed whether the First-Brain Perspective represents a mere conjunction where both perspectives are purely linked to each other. Or whether it is a genuinely unifying ground underlying and necessarily entailing first- and third-person perspective. In the latter case the First-Brain Perspective would need to be characterized by states different from both neuronal and mental states since otherwise it would not be genuinely unifying. This may be something like Nagel envisions when he speaks of the "right point of view" The right point of view (i.e. First-Brain Perspective) would be one which, contrary to present conceptual possibilities, included both subjectivity and spatiotemporal structure from the outset, so that it would describe inner states and their functional relations to behavior from the phenomenological inside and the physiological outside simultaneously – not in parallel. "The mental and physiological concepts and their reference to this same inner phenomenon would then be seen as secondary and each partial in its grasp of the phenomenon: each would be seen as referring to something that extends beyond its ground of application" [23]. It is clear that the description of the First-Brain Perspective given here in the empirical context of first-person perspective falls far short from the conceptual requirements postulated by Nagel. Therefore, the presented concept of First-Brain Perspective may be considered, if at all, a starting point, which needs further conceptual elaboration, especially with regard to the various philosophical implications.
